# Oestrogen-receptor status and endocrine therapy of breast cancer: response rates and status stability.

**DOI:** 10.1038/bjc.1981.8

**Published:** 1981-01

**Authors:** R. E. Leake, L. Laing, K. C. Calman, F. R. Macbeth, D. Crawford, D. C. Smith

## Abstract

The concentration of cellular oestrogen receptor (RE) was measured in both the soluble and nuclear-pellet fractions of biopsies from 1,000 breast cancers. Data suggest that functional steroid RE is always in equilibrium between the soluble and nuclear fractions. However, biopsies from only one-third of patients contained detectable amounts of high-affinity RE in both fractions. Thirty patients out of 42 (71%) whose biopsies contained RE in both fractions, showed objective remission after receiving some form of hormonal manipulation as sole treatment. Response rates in the other categories ranged from 9% for those whose biopsies contained no detectable RE to 24% for those who displayed soluble RE alone. The presence of RE in both fractions of primary disease, whereas RE-negativity was maintained during progression from primary to secondary disease. Other aspects of RE status in relation to stage of disease are analysed.


*Br. J. Cancer* (1981) **43**, 59

### OESTROGEN-RECEPTOR STATUS AND ENDOCRINE THERAPY OF BREAST CANCER: RESPONSE RATES AND STATUS STABILITY

R. E. LEAKE*, L. LAING*, K. C. CALMAN†, F. R. MACBETH*†, D. CRAWFORD‡ AND D. C. SMITH‡

*From the Departments of *Biochemistry and †Oncology, Glasgow University and the ‡Department of Surgery, Victoria Infirmary, Glasgow*

Received 8 May 1980 Accepted 30 September 1980

**Summary.—The concentration of cellular oestrogen receptor (RE) was measured in both the soluble and nuclear-pellet fractions of biopsies from 1,000 breast cancers. Data suggest that functional steroid RE is always in equilibrium between the soluble and nuclear fractions. However, biopsies from only one-third of patients contained detectable amounts of high-affinity RE in both fractions. Thirty patients out of 42 (71%) whose biopsies contained RE in both fractions, showed objective remission after receiving some form of hormonal manipulation as sole treatment. Response rates in the other categories ranged from 9% for those whose biopsies contained no detectable RE to 24% for those who displayed soluble RE alone. The presence of RE in both fractions of primary disease was found to be an unreliable index of RE status in subsequent secondary disease, whereas RE-negativity was maintained during progression from primary to secondary disease. Other aspects of RE status in relation to stage of disease are analysed.**

ENDOCRINE THERAPY is a long-established treatment of secondary breast cancer (Beatson, 1896). It is, however, successful in only a small (~25%) proportion of cases (King & Roberts, 1979). The choice of endocrine therapy for a particular patient has been made on the basis of several clinical features, such as menopausal status, disease-free interval, site of dominant lesion, *etc.*, and the response to any earlier endocrine therapy (Pearson & Ray, 1960; McGuire *et al.*, 1977). The absence of a single reliable index of hormone dependence of breast tumours has led to a marked decrease in recent years of the use of ablative therapies.

Preliminary data from our laboratory (Laing *et al.*, 1977) have already indicated that, in patients with advanced breast cancer, response to endocrine therapy was more likely when the tumour contained oestrogen receptor RE in both the soluble and pellet fractions. The same data revealed the existence of oestrogen receptor ($RE_N$) in the pellet, in the absence of any soluble receptor ($RE_C$), a previously unconsidered possibility. This study also raised the question of the existence of $RE_N$ that was either unfilled by steroid or, alternatively, bound to chromatin in a manner which allowed the steroid to dissociate at low temperatures.

The present paper reports both soluble and pellet RE status for 1000 patients. It then analyses the breakdown of RE status in relation to menopausal and nodal status and to stage of disease. Responses to endocrine therapy of 129 patients with advanced disease in relation to RE status is also reported.

Given the value of RE status as a tool in determining therapy for secondary

Address for correspondence: Dr R. E. Leake, Department of Biochemistry, University of Glasgow, Glasgow G12 8QQ.

disease, it would be very useful if such status could be shown to reflect that in primary disease. This would be particularly valuable in cases in which secondary disease is surgically inaccessible. Similarly, the maintenance of RE status between early (often local) and later recurrences is also of considerable interest. RE status in biopsies of both primary and secondary disease is reported for 32 patients and corresponding data between early and later recurrence for 20 patients.

### MATERIALS AND METHODS

### *Materials*

$^3$H-oestradiol-17$\beta$ (sp. act. 44 mCi/$\mu$mol) was obtained from The Radiochemical Centre, Amersham, England.

All reagents were AnalaR grade.

Solutions were prepared in glass-distilled water, since the presence of metal ions was found to interfere with the assay of receptors.

Human breast tumour tissue was obtained from 8 hospitals in the Glasgow area.

### *Methods*

*Tissue fractionation.*—Tissue was collected fresh and transported from the operating theatre to the laboratory on ice. Wherever possible, RE assay was performed the same day, but, when this could not be achieved, storage was at −20°C in sucrose buffer (0·25M sucrose, 1·5mM $MgCl_2$, 10mM Hepes, pH 7·4)/50% glycerol (v:v) (Leake *et al.*, 1979). Soluble and nuclear fractions were then prepared as follows.

About 150 mg of tissue was dissected from the area adjacent to that removed for pathological examination. Homogenization was carried out at 50 mg/ml in 10mM Hepes, 1·5mM EDTA, 0·25mM DTT, pH 7·4 (HED buffer) using 2 × 10s bursts at a setting of 150 on an Ultra-Turrax, model TP 18/2, followed by further homogenization with a glass tissue grinder (Kontes Duall). The homogenate was centrifugated at 5000 *g* for 5 min at 4°C to yield a "cytosol" supernatant and a crude nuclear pellet. The pellet was washed ×3 in 0·15M NaCl, 10mM Hepes (pH 7·4), and finally resuspended to the original volume in buffered saline. A wash with 0·1% Triton X-100 was on occasion incorporated at this stage to further purify the nuclear material, but this did not appreciably alter the level of nuclear binding. Further purification of the pellet fraction by differential centrifugation through sucrose (finally 2·4M sucrose, 1·5mM $MgCl_2$) did not significantly alter RE content expressed per unit DNA.

*Assay of receptors.*—The initial procedures in the assay system were identical for both tissue fractions. 150$\mu$l aliquots of cytosol or nuclear suspension were added to 50$\mu$l aliquots of $^3$H-oestradiol-17$\beta$ to give final concentrations of steroid of 1, 1·5, 2, 4, 6 and $8 \times 10^{-10}$M. Two additional tubes were also set up containing $10^{-9}$M $^3$H-oestradiol with or without $10^{-7}$M unlabelled diethylstilboestrol (DES) to determine the specificity of binding. All tubes were then incubated at 4°C for 18 h. The inclusion of protease inhibitors Trasylol and/or phenylmethylsulphonyl fluoride (PMSF) in the incubation medium did not appear to enhance RE measurement. After incubation, the amount of steroid bound was determined for each fraction as follows.

*Cytosol receptors* ($RE_C$).—At the end of the incubation period, 0·9 ml of 1·5mM EDTA, 10mM Hepes (pH 7·4) and 0·5 ml Dextran-coated charcoal (0·15% (w:v) charcoal and 0·0015% (w:v) dextran T-70, equilibrated in 0·25M sucrose, 1·5mM EDTA, 10mM Hepes, pH 7·4) were added to each tube. This mixture was agitated at 0°C for 15 min followed by centrifugation at 1000 *g* for 5 min. To 1ml aliquots of each supernatant was added 10 ml Triton–toluene scintillant (200 ml ethanol:600 ml Triton X-100:1400 ml toluene/PPO (5 g/l)/POPOP (0·24 g/l)) and each counted at 25% efficiency in a Philips or at 30% in a Searle Mark III Liquid Scintillation analyser.

*Nuclear receptors* ($RE_N$).—Following incubation, 100$\mu$l aliquots were removed from each tube and added to 5ml saline. This mixture was poured down the chimney of a Millipore filter apparatus on to a pre-wetted Whatman GF/C glass-fibre filter. The tube was washed out with 5 ml saline, the washing poured on to the filter and the filter further washed with 3 × 4 ml saline under suction. After removal of the chimney, the edge of the filter was washed and the filter removed into a scintillation vial prior to drying overnight at 60°C. 10 ml toluene/PPO (5 g/l) scintillant was added, and the samples counted at 35%

efficiency in a Philips or Searle Mark III Liquid Scintillation analyser.

*Protein and DNA assay.*—Cytosol protein concentration was determined by the method of Lowry.

DNA content was determined by a modification of the method of Burton (1956) as described by Katzenellenbogen & Leake (1974).

*Definition of positivity*

To be classed as $RE^+$ the binding displayed by either tissue fraction was required to fulfil 3 criteria: (a) yield an unambiguous Scatchard plot, which produces (b) a straight line, giving a $K_d$ in the range $0{\cdot}5$–$5 \times 10^{-10}$M; (c) specificity must be established by competition with excess diethylstilboestrol. RE concentrations as low as 3 fmol/mg protein and 25 fmol/mg DNA were detected for the soluble and pellet fractions respectively.

Response to hormone therapy was assessed in patients with secondary disease for whom (a) RE status had been determined before the initiation of any therapy, (b) endocrine therapy alone was applied as first treatment during the period of assessment. The criteria for response were those suggested by the British Breast Group (1974). In brief, these involve at least 50% regression of existing lesions, and no appearance of new lesions within a 6-month period. Only patients satisfying these criteria for at least 6 months are recorded as having responded (Table VII).

### RESULTS

*Primary disease*

The distribution of patients by RE status is shown in Table I. This is a compilation of data from pre- or post-menopausal patients with primary disease. Patients with RE in both soluble and pellet fractions are classified as (+/+), those with only $RE_C$ as (+/0), those with only $RE_N$ as (0/+) and those with RE in neither fraction as (0/0).

Tumours with functional oestrogen RE would be expected to display both $RE_C$ and $RE_N$, even at very high plasma oestrogen levels, since an equilibrium is always maintained between filled receptor in the 2 pools (Williams & Gorski, 1971; Sheridan *et al.*, 1979). Patients in the +/+ category would, therefore, be expected to have hormone-sensitive tumours, whereas all other categories of tumour might be expected to be autonomous, or respond to endocrine therapy only by an indirect route.

TABLE I.—*Analysis of cytoplasmic and nuclear oestrogen receptors in* 1000 *biopsies of breast tumour tissue*

| Receptor content $RE_C/RE_N$ | No. patients | % |
|---|---|---|
| +/+ | 343 | 34 |
| 0 /0 | 479 | 48 |
| +/0 | 118 | 12 |
| 0/+ | 60 | 6 |

When RE status of patients is reanalysed in relation to menopausal status (Table II) it is seen that the proportions in each of the categories (1) functional $RE^+$, (2) $RE^-$ and (3) $RE_C$ alone remain fairly similar. However, the small group of tumours which contain only $RE_N$ appears to be confined to post-menopausal patients. This suggests either an abnormality in RE function associated with menopause or a failure to exchange oestrogen on to this class of receptor in the pre-menopausal nuclear samples under the conditions used. Since the $RE_N$ in +/+ samples of premenopausal patients clearly does exchange oestrogen, the latter suggestion is perhaps less likely.

TABLE II.—*Distribution of oestrogen receptors between the cytoplasmic and nuclear fractions of breast tumour tissue from pre- and post-menopausal patients*

| $RE_C/RE_N$ | Premenopausal No. patients (%) | Postmenopausal No. patients (%) |
|---|---|---|
| +/+ | 22 (32) | 69 (36) |
| 0/0 | 34 (50) | 81((42) |
| +/0 | 12 (18) | 26 (13) |
| 0/+ | 0 (0) | 17 (9) |

Analysis of RE status of primary disease in relation to stage of disease is shown in Table III. The number of patients involved in each individual category is fairly small. There is no significant difference in the stage of the disease at

TABLE III.—*Comparison of RE status and clinical stage in biopsies of 191 primary breast cancers*

| | No. patients in each stage | | | |
|---|---|---|---|---|
| $RE_C/RE_N$ | I | II | III | IV |
| +/+ | 26 | 27 | 5 | 1 |
| 0/0 | 38 | 55 | 6 | 4 |
| +/0 | 7 | 7 | 0 | 1 |
| 0/+ | 6 | 8 | 0 | 0 |

first presentation when biopsies are classified as containing functional RE (+/+) or completely lacking in $RE_C$ (0/0). This is, perhaps, surprising in view of the concept that absence of RE indicates a more rapidly progressing tumour (Meyer *et al.*, 1977).

The distribution of RE was also reanalysed in relation to nodal status. It was thought that patients with $RE^-$ tumours would be more likely to exhibit nodal involvement than those with $RE^+$ disease. However, the data in Table IV do

TABLE IV.—*Comparison of RE status and nodal status in 134 breast tumour biopsies*

| | No. patients | |
|---|---|---|
| $RE_C/RE_N$ | Node −ve (%) | Node +ve (%) |
| +/+ | 20 (49) | 21 (51) |
| 0/0 | 26 (40) | 39 (60) |
| +/0 | 5 (33) | 10 (67) |
| 0/+ | 8 (62) | 5 (38) |

not support this idea. The potential for nodal infiltration is clearly not dependent on receptor status. This observation agreed with that of Hähnel *et al.* (1979) although a loose relationship between nodal involvement and RE-negativity was reported by Allegra *et al.* (1979).

*Receptor status stability*

Much of the early interest in RE status was derived from the idea that measurements on biopsies of primary disease would act as reliable therapeutic indices once secondary growth was detected (King, 1975; Jensen, 1975). However, practical demonstration of the stability of RE status between biopsy and the appear-

TABLE V.—*$RE_C$ and $RE_N$ status in biopsies of primary and secondary breast disease from the same patient*

| Patient | Age | Months between biopsies | $RE_C/RE_N$ status: Primary | Secondary |
|---|---|---|---|---|
| 517535 | 59 | 34 | 0/0 | 0/0 |
| 529941 | 41 | 11 | 0/0 | 0/0 |
| 490197 | 64 | 19 | +/+ | +/+ |
| 543284 | 58 | 19 | 0/0 | 0/0 |
| 206073 | 40 | 10 | 0/0 | 0/0 |
| 335662 | 56 | 17 | +/+ | 0/0 |
| 554902 | 78 | 8 | 0/+ | +/0 |
| 517288 | 59 | 8 | +/+ | +/+ |
| 512434 | 44 | 14 | 0/0 | 0/0 |
| 338381 | 52 | 21 | +/0 | 0/+ |
| 190658 | unknown | 13 | +/0 | 0/+ |
| 533839 | 38 | 21 | 0/0 | 0/0 |
| 550668 | 43 | 31 | 0/0 | 0/+ |
| 559665 | 64 | 7 | +/0 | 0/0 |
| 528446 | 68 | 16 | +/+ | +/+ |
| 528171 | 68 | 2 | 0/0 | 0/0 |
| 297738 | 46 | 11 | 0/0 | 0/0 |
| 519488 | 49 | 12 | 0/0 | 0/0 |
| 420564 | 52 | 21 | 0/0 | +/+ |
| 348637 | 57 | 5 | 0/0 | 0/0 |
| 525191 | 77 | 12 | 0/0 | 0/0 |
| 498101 | 43 | 6 | 0/0 | 0/0 |
| 227746 | 75 | 23 | 0/0 | 0/0 |
| 551907 | 64 | 12 | +/+ | +/+ |
| 263806 | 80 | 4 | 0/0 | 0/0 |
| 526290 | 44 | 19 | +/+ | +/0 |
| 341527 | 52 | 5 | 0/0 | 0/0 |
| 518777 | 49 | 23 | +/+ | 0/0 |
| 297515 | 71 | 18 | +/+ | +/0 |
| 305543 | 67 | 15 | 0/+ | +/0 |
| 409965 | 46 | 32 | +/+ | +/+ |
| 653306 | unknown | 23 | +/+ | 0/+ |

ance of secondary disease has been limited due to the difficulties in (a) maintaining a stable patient population and (b) obtaining sufficient material from the metastatic site. Table V shows RE status determined in primary and subsequent secondary disease from 32 patients. None of the patients received known relevant therapy in the intervening period. In 20 cases (63%) RE status is the same for both biopsies. Only 5/10 receptor-positive cases remained +/+ indicating that hormone dependence in primary disease is not necessarily retained in secondary disease. Only one out of 17 $RE^-$ patients (0/0) developed $RE^+$ (+/+) secondary growth. Both tamofixen (Leake *et al.*, 1979) and chemotherapy have been found to either block RE synthesis or interfere with the RE assay, but this patient had received no such relevant therapy prior to biopsy.

Thus a $RE^-$ primary is almost certain to give rise to hormone insensitive secondary disease.

When RE status is compared between first occurrence and later recurrences (Table VI) it is again clear that 0/0 disease generally retains this status. Of 12 biopsies examined, only one changed status. Once more, it was striking that change of status was common in biopsies with RE in only one fraction. Of the $RE^+$ biopsies obtained in early recurrence, 3/4 retained functional RE (+/+).

Further examination of the group of patients whose biopsies had $RE_C$ alone was carried out. It was apparent (Figure) that the RE concentration in tissues with $RE_C$ alone (+/0) was relatively lower than that in $RE^+$ (+/+) biopsies. However, a significant number of biopsies (11/118) in this category (+/0) had receptor concentrations in excess of 100 fmol/mg protein. Thus, although there is an indication that high $RE_C$ concentration is equivalent to a good chance of response to endocrine therapy, no absolute rule applies.

FIGURE.—The concentration of soluble RE in biopsies from patients with functional RE (+/+, □) and those with RE only in the soluble fraction (+/0, ■). The total number of biopsies in each of the two categories (118) was identical.

TABLE VI.—*$RE_C$ and $RE_N$ status in biopsies of more than one secondary deposit from the same breast-cancer patient*

| Patient | Age at first biopsy | Months between biopsies | $RE_C/RE_N$ 1st sample | 2nd sample | 3rd sample |
|---|---|---|---|---|---|
| 529941 | 41 | 2 | 0/0 | 0/0 | — |
| 503664 | 48 | 13 & 9 | 0/0 | 0/0 | 0/0 |
| 543284 | 58 | 4 | 0/0 | 0/0 | — |
| 612828 | unknown | 23 | +/0 | 0/0 | — |
| 517288 | 59 | 14 & 10 | +/+ | +/+ | 0/0* |
| 576240 | 47 | 18 | 0/0 | 0/0 | — |
| 190658 | unknown | 6 | 0/+ | +/+ | — |
| 416889 | 60 | 14 | 0/0 | 0/0 | — |
| 297738 | 46 | 6 | 0/0 | +/+ | — |
| 560179 | 64 | 10 | 0/0 | 0/0 | — |
| 519488 | 49 | 7 | 0/0 | 0/0 | — |
| 420564 | 52 | 1 | +/+ | +/0 | — |
| 482442 | 49 | 11, 12 & 2 | +/0 | 0/0 | +/+† |
| 525191 | 77 | 7 | 0/0 | 0/0 | — |
| 249687 | unknown | 10 | 0/0 | 0/0 | — |
| 170263 | 72 | 25 | 0/0 | 0/0 | — |
| AF/V | unknown | 14 | +/+ | +/+ | — |
| 526290 | 44 | 10 | +/0 | 0/0 | — |
| 518777 | 49 | 3 | 0/0 | 0/0 | — |
| 544403 | 45 | 30 | +/+ | +/+ | |

* Patient withdrawn from tamoxifen only 10 days previously (see text).

† Fourth biopsy—0/0.

*Endocrine therapy of advanced disease*

All patients with advanced disease for whom the RE status of an appropriate biopsy was known, were monitored throughout subsequent treatment. The response of those patients who received any type of hormonal therapy as first-line treatment for any period was noted in relation to the criteria listed earlier. The results are summarized in Table VII. Patients whose biopsies showed an intact RE system had a very good chance of responding to some type of endocrine therapy (most commonly tamoxifen treatment). Only 5 patients (9%) of those in the truly $RE^-$ class showed good response. In each case these patients had received tamoxifen, and may have responded to one of the actions of this drug which is not RE-mediated (Tisdale, 1977). It is

TABLE VII.—*Response of breast tumours to hormone therapy in relation to their RE content*

| $RE_C/RE_N$ | Total patients | Complete response (%) |
|---|---|---|
| +/+ | 45 | 32 (71) |
| 0/0 | 58 | 5 (9) |
| +/0 | 17 | 4 (24) |
| 0/+ | 9 | 1 (11) |

striking that the patients whose biopsies contained RE in only one fraction (0/+ or +/0) behaved in a manner similar to the $RE^-$ group, suggesting that these receptors are non-functional, though there is no indication whether the fault lies in the RE itself or in some cellular recognition site.

Of the patients in Table VII, those who did not experience a complete response for 6 months were divided as follows: in the (+/+) category 8 had progressive disease, 1 was static and 3 showed a partial response; in the (0/0) category 48 had progressive disease, 3 were static and 2 showed a partial response; in the (+/0) category 10 had progressive disease, 1 was static and 2 showed partial response; in the (0/+) category all 8 patients had progressive disease. Of the 129 patients considered, only 27 were pre-menopausal and 10 menopausal. The response rates quoted, therefore, apply principally to post-menopausal disease. It is, however, significant that 18/27 pre-menopausal patients had both biopsies with no detectable RE (0/0) and suffered progressive disease. Of the 42 patients experiencing complete response, 18 had local recurrence, 10 had recurrence in gland and/or skin, 7 in bone and the remainder at one or more distant sites. Of 74 patients with progressive disease, 13 had local recurrence, 21 skin and/or gland, 21 bone, 7 pleura, 7 liver and the remainder at one or more distant sites.

### DISCUSSION

Both established dogma (Leake, 1976) and recent interpretations (Sheridan *et al.*, 1979) of steroid hormone action essentially require that functional hormone RE complex forms an equilibrium between the soluble and nuclear-pellet fractions of the cell. Such an equilibrium is rapidly established at 37°C, but can also be established at 0°C over 22 h (Traish *et al.*, 1979). Similarly, the distribution of RE between the soluble and nuclear-pellet fractions of target tissue has also been successfully measured at both 37°C and 4°C by use of different incubation times, though the decreased stability of receptor at 37°C in the cell-free environment meant that assay at 4°C (or 20°C) gave more reproducible results (Leake *et al.*, 1979). Thus, hormonal dependence of a particular human breast tumour biopsy should be reflected in the presence of measurable quantities of RE in both soluble and pellet fractions of said biopsy.

After adopting strict criteria for the measurement of cellular RE (Leake *et al.*, 1979) it was found that only one-third of patients with primary disease yielded biopsies containing functional RE (Table I), *i.e.* RE in both soluble and pellet fractions. Biopsies from about half the patients had undetectable levels of high-affinity RE. This is a surprisingly large proportion, but has been maintained throughout the study. Further, the low rate of response of advanced disease to hormone therapy in patients lacking RE (Table VII), taken together with the observation that $RE^-$ (0/0) primaries give rise to $RE^-$ secondaries (Table V), suggest that such a high incidence of hormonal insensitivity is real. The 2 initially unexpected groups of patients (+/0) and (0/+) (Laing *et al.*, 1977) continue to present. Such patients with RE in one fraction of the biopsy only have now also been observed in other studies (Panko & MacLeod, 1978; Thorsen, 1979; Barnes *et al.*, 1979).

Much of the value of determining RE status in primary disease depends upon the assumption that in subsequent secondary disease RE status will faithfully reflect that in the primary biopsy. However, in a study of 32 patients for whom RE status was determined in both primary and advanced disease (Table V), only half the primaries with fully functional RE gave rise to (+/+) secondaries. This is a disappointingly low level of consistency of RE status between primary and secondary disease, but may reflect the fact that the secondary samples are necessarily selected from surgically accessible sites. The consistency of RE status might be higher if all sites of secondary disease were considered. These patients had received no adjuvant therapy, so the loss of RE must have resulted during the natural progression of the disease. Further studies in progress may clarify this situation.

It was more encouraging to find that RE status in only 1 patient out of 17 reverted from $RE^-$ primary to fully $RE^+$. Patients whose receptors fell in the abnormal categories (+/0 or 0/+) were found to show a high level of variation between primary and secondary disease. However, there were no cases of change to $RE^+$ status. Hence patients whose primaries are either $RE^-$ or abnormal have very little chance of subsequently responding to hormonal manipulation.

The follow-up data in Table VII show that patients whose biopsies of secondary disease contain fully functional RE have a much better chance of objective response to human manipulation than do those with either no RE or RE in one fraction only. The criteria of clinical response used in this paper are quite severe (British Breast Group, 1974) similar to those proposed by the UICC (Hayward *et al.*, 1977). Stoll (1977) proposed shorter periods of sustained response. Adoption of less stringent criteria will increase the response rate in any series. However, no biological index is ever likely to identify potential responders with complete accuracy, since so many variables are involved. Alternative indices of hormonal-dependence have been tried, and perhaps the most successful is measurement of soluble progesterone receptor, a product of oestrogen action in normal target tissue. Recent studies by Barnes *et al.* (1979), Thorsen & Stoa (1979) and in our own laboratory suggest that although the presence of RP is not always associated with an improved clinical response, it is usually associated with the presence of fully functional RE and so yields a similar success rate in the identification of responders to hormone therapy.

We are extremely grateful to the Cancer Research Campaign whose financial assistance has been essential to this study. We should also like to thank Professor R. M. S. Smellie for his provision of facilities and also for his helpful comments and criticisms. We are very pleased to acknowledge the co-operation of many of our surgical colleagues, especially Mr Frank Crossling, Mr Colin McArdle and Mr John Maxwell Anderson.

### REFERENCES

ALLEGRA, J. C., LIPPMAN, M. E., THOMPSON, E. B. & 6 others (1979) Distribution, frequency and quantitative analysis of estrogen, progesterone, androgen and glucocorticoid receptors in human breast cancer. *Cancer Res.*, **39**, 1447.

BARNES, D. M., SKINNER, L. G. & RIBEIRO, G. G. (1979) Triple hormone-receptor assay: A more accurate predictive tool for the treatment of advanced breast cancer? *Br. J. Cancer*, **40**, 682.

BEATSON, G. T. (1896) On the treatment of inoperable cases of carcinoma of the mamma: Suggestions for a new method of treatment with illustrative cases. *Lancet*, ii, 162.

BRITISH BREAST GROUP (1974) Assessment of response to treatment in advanced breast cancer. *Lancet*, ii, 38.

BURTON, K. (1956) A study of the conditions and mechanisms of diphenylamine reactions for the colorimetric estimation of deoxyribonucleic acid. *Biochem. J.*, **62**, 315.

HÄHNEL, R., WOODINGS, T. & VIVIAN, A. B. (1979) Prognostic value of estrogen receptors in primary breast cancer. *Cancer*, **44**, 671.

HAYWARD, J. L., CARBONE, P. P., HEUSON, J. C., KUMAOKA, S., SEGALOFF, A. & RUBENS, R. D. (1977) Assessment of response to therapy in advanced breast cancer. *Eur. J. Cancer*, **13**, 89.

JENSEN, E. V. (1975) Estrogen receptors in hormone-dependent breast cancers. *Cancer Res.*, **35**, 3362.

KATZENELLENBOGEN, B. S. & LEAKE, R. E. (1974) Distribution of the oestrogen-induced protein and of total protein between endometrial and myometrial fractions of the immature and mature rat uterus. *J. Endocrinol.*, **63**, 439.

KING, R. J. B. (1975) Clinical relevance of steroid-receptor measurements in tumours. *Cancer Treat. Rev.*, **2**, 273.

KING, R. J. B. & ROBERTS, M. M. (1979) The use of steroid receptor assays in predicting response to endocrine therapy: A summary of the clinical data. In *Steroid Receptor Assays in Human Breast Tumours: Methodological and Clinical Aspects.* Ed. King. Cardiff: Alpha Omega. p. 1.

LAING, L., SMITH, M. G., CALMAN, K. C., SMITH, D. C. & LEAKE, R. E. (1977) Nuclear oestrogen receptors and treatment of breast cancer. *Lancet*, ii, 168.

LEAKE, R. E. (1976) Current views on oestrogen receptors. *Trends Biochem. Sci.*, **1**, 137.

LEAKE, R. E., LAING, L. & SMITH, D. C. (1979) A role for nuclear oestrogen receptors in prediction of therapy regime for breast cancer patients. In *Steroid Receptor Assays in Human Breast Tumours: Methodological and Clinical Aspects.* Ed. King. Cardiff: Alpha Omega. p. 73.

MCGUIRE, W. L., HORWITZ, K. B., PEARSON, D. H. & SEGALOFF, A. (1977) Current status of estrogen and progesterone receptors in breast cancer. *Cancer*, **39**, 2934.

MEYER, J. S., RAO, B. R., STEVENS, S. C. & WHITE, W. L. (1977) Low incidence of estrogen receptor in breast carcinomas with rapid rates of cellular replication. *Cancer*, **40**, 2290.

PANKO, W. B. & MACLEOD, R. M. (1978) Uncharged nuclear receptors for estrogen in breast cancers. *Cancer Res.*, **38**, 1948.

PEARSON, O. H. & RAY, B. S. (1960) Hypophysectomy in the treatment of metastatic mammary cancer. *Am. J. Surg.*, **99**, 544.

SHERIDAN, P. J., BUCHANAN, J. M., ANSELMO, V. C. & MARTIN, P. M. (1979) Equilibrium: The intracellular distribution of steroid receptors. *Nature*, **282**, 579.

STOLL, B. A. (1977) "False-positive" oestrogen-receptor assay in breast cancer. *Lancet*, ii, 296.

THORSEN, T. (1979) Occupied and unoccupied nuclear oestradiol receptor in human breast tumours: Relation to oestradiol and progesterone cytosol receptors. *J. Steroid Biochem.*, **10**, 661.

THORSEN, T. & STOA, K. F. (1979) Nuclear uptake of oestradiol-17$\beta$ in human mammary tumour tissue. *J. Steroid Biochem.*, **10**, 595.

TISDALE, M. J. (1977) Inhibition of prostaglandin synthetase by anti-tumour agents. *Chem. Biol. Interact.*, **18**, 91.

TRAISH, A. M., MULLER, R. E. & WOTIZ, H. H. (1979) Comparison of formation, activation and nuclear translocation of receptor–oestradiol ($R \cdot E_2$) complex at 0°C and 37°C in intact uterine cells. *J. Biol. Chem.*, **254**, 6560.

WILLIAMS, D. & GORSKI, J. (1971) A new assessment of subcellular distribution of bound estrogen in the uterus. *Biochem. Biophys. Res. Commun.*, **45**, 258.


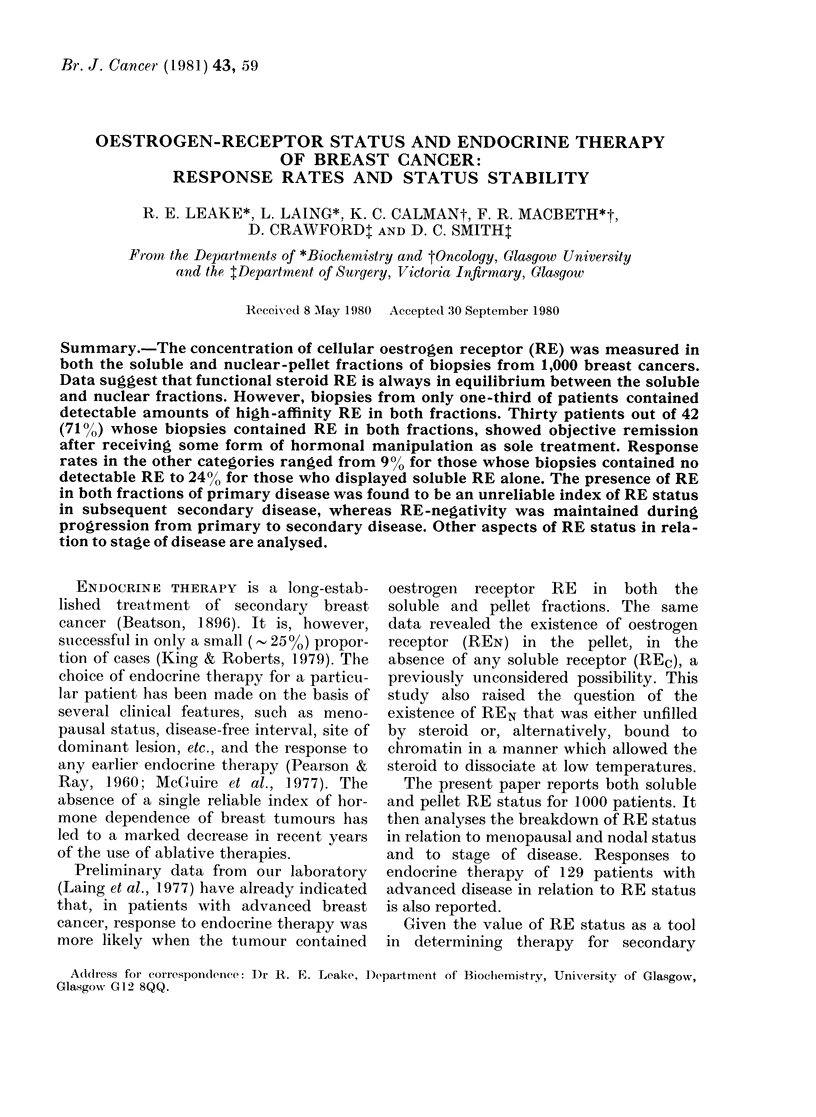

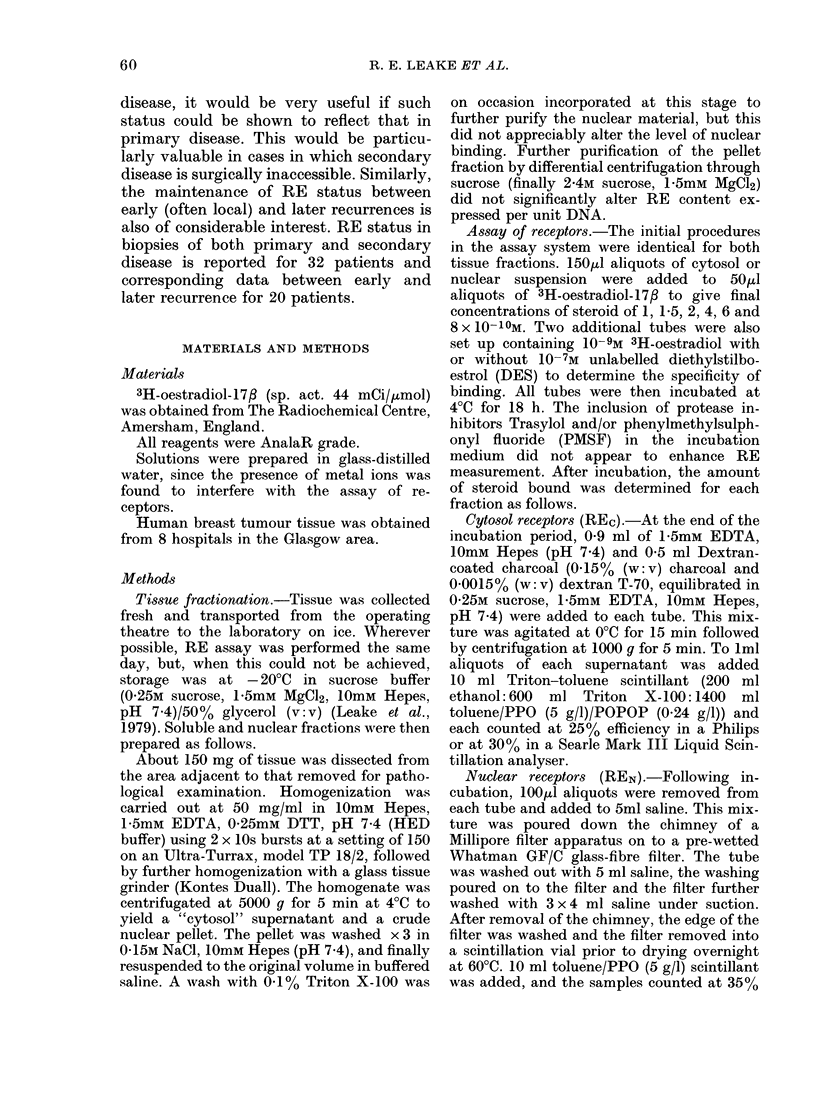

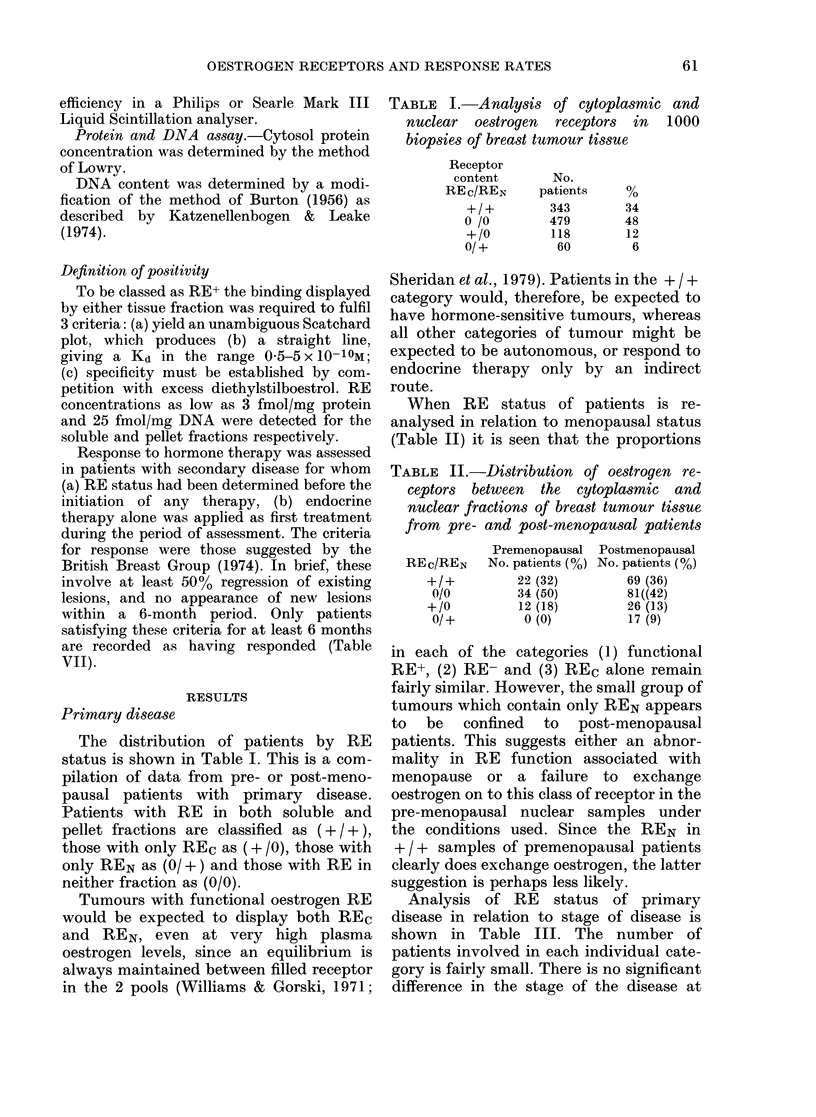

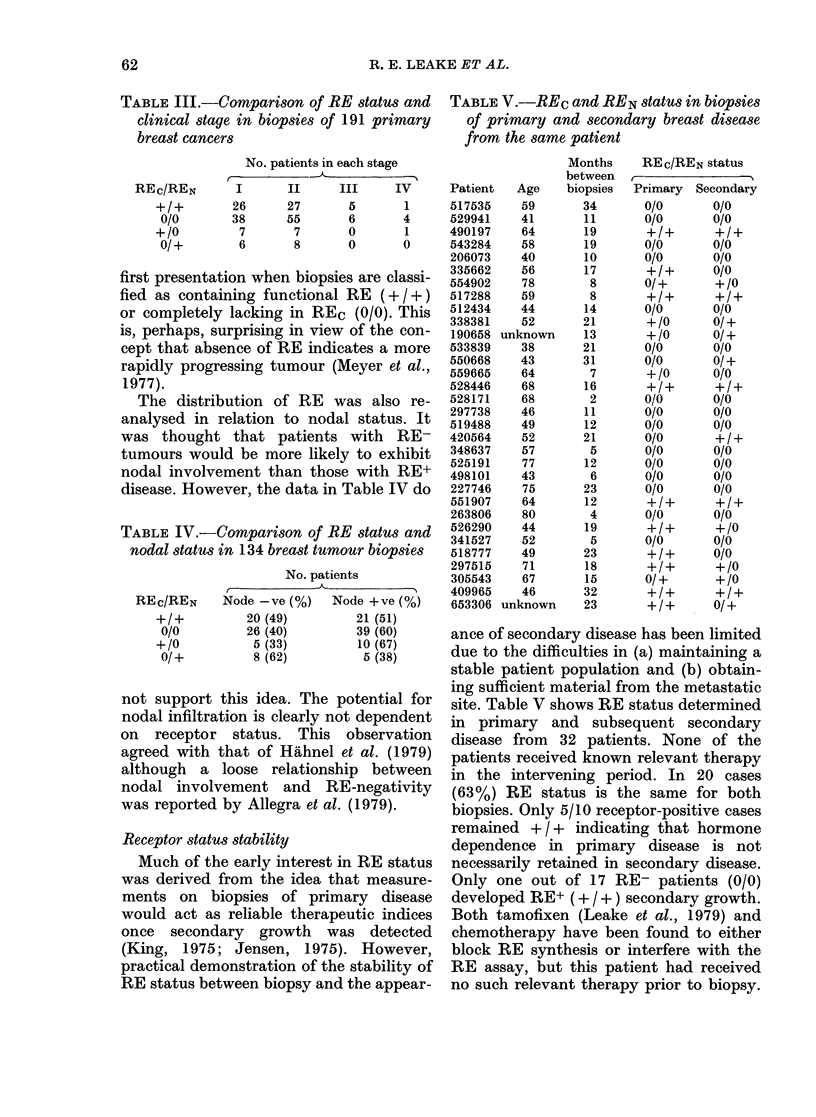

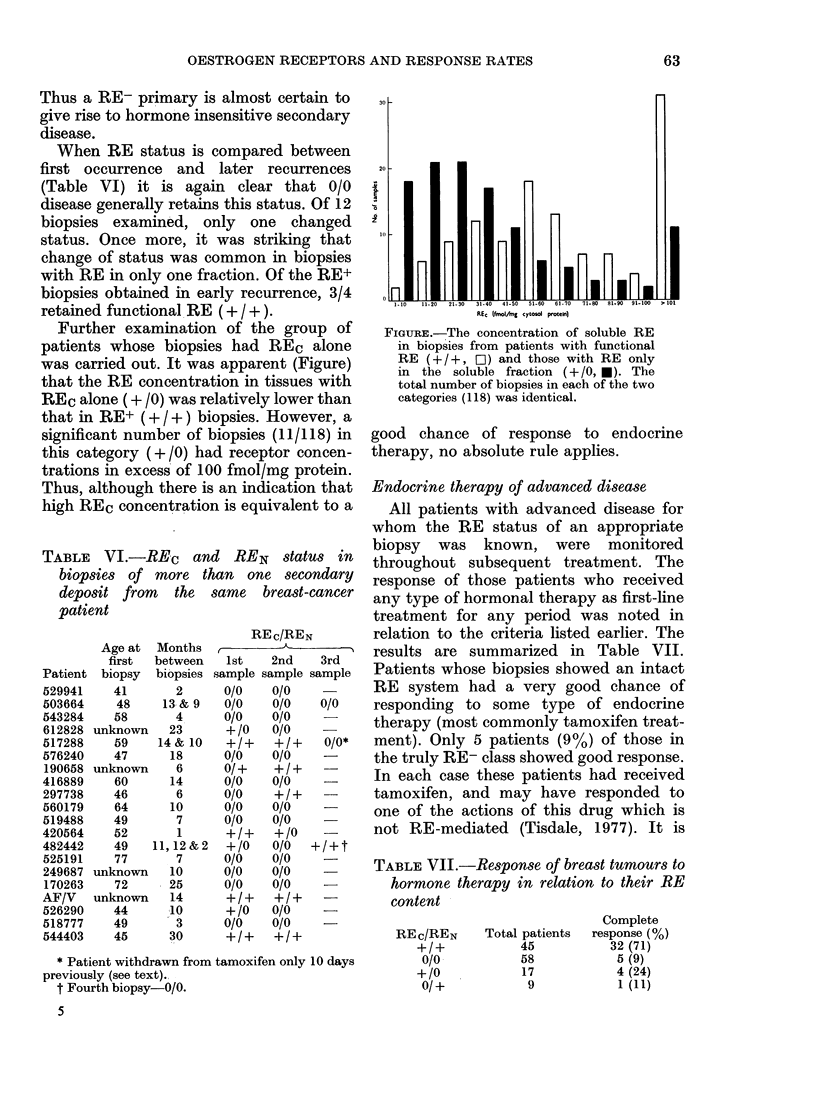

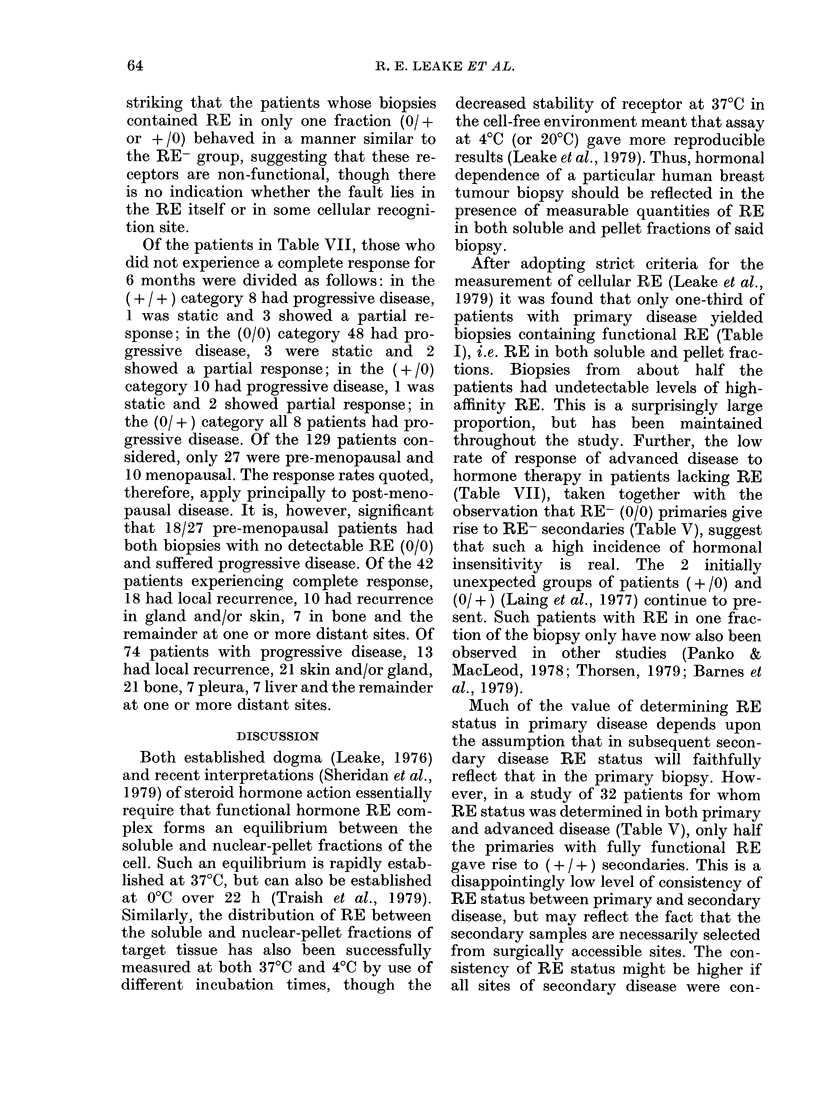

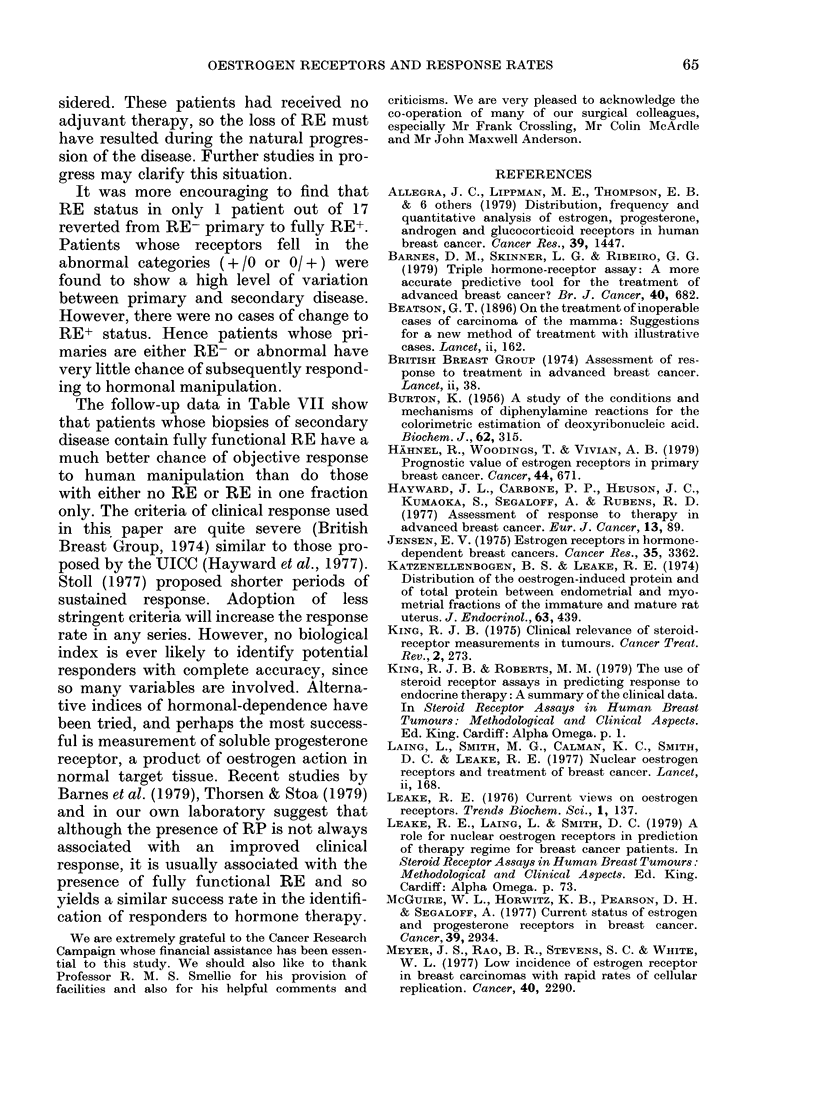

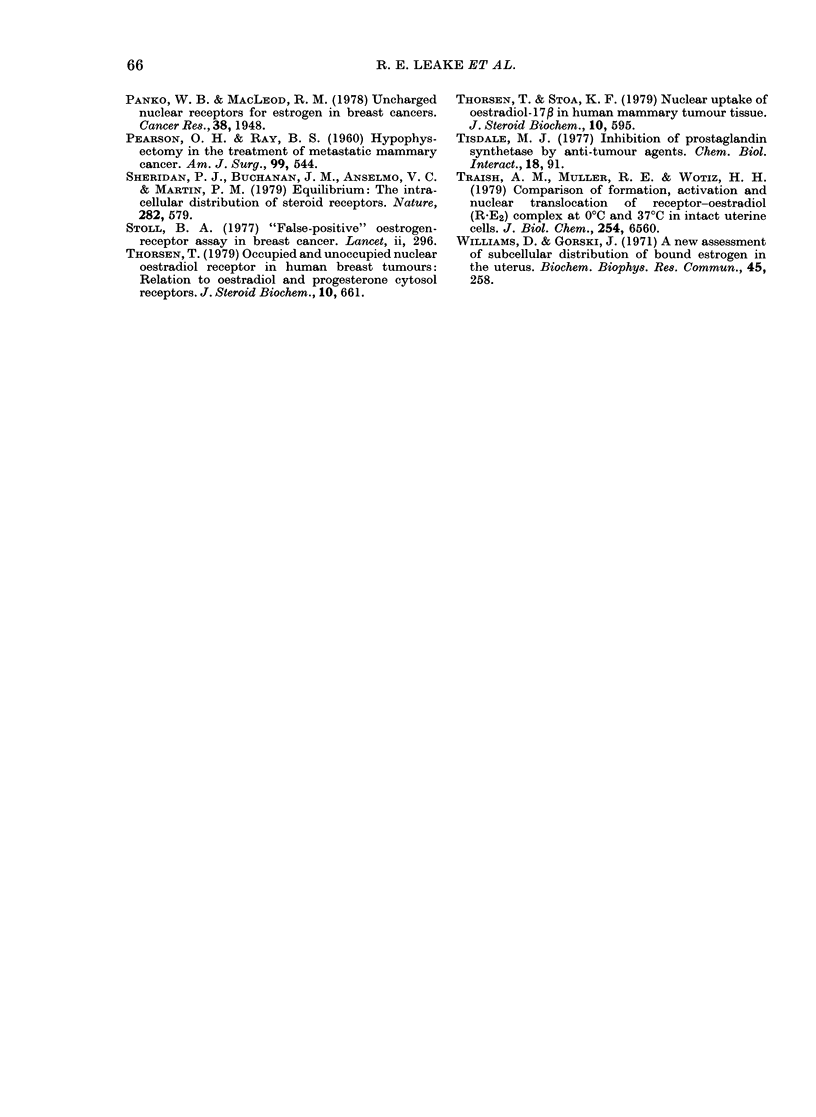


## References

[OCR_00971] Allegra J. C., Lippman M. E., Thompson E. B., Simon R., Barlock A., Green L., Huff K. K., Do H. M., Aitken S. C. (1979). Distribution, frequency, and quantitative analysis of estrogen, progesterone, androgen, and glucocorticoid receptors in human breast cancer.. Cancer Res.

[OCR_00994] BURTON K. (1956). A study of the conditions and mechanism of the diphenylamine reaction for the colorimetric estimation of deoxyribonucleic acid.. Biochem J.

[OCR_01000] Hähnel R., Woodings T., Vivian A. B. (1979). Prognostic value of estrogen receptors in primary breast cancer.. Cancer.

[OCR_01011] Jensen E. V. (1975). Estrogen receptors in hormone-dependent breast cancers.. Cancer Res.

[OCR_01014] Katzenellenbogen B. S., Leake R. E. (1974). Distribution of the oestrogen-induced protein and of total protein between endometrial and myometrial fractions of the immature and mature rat uterus.. J Endocrinol.

[OCR_01021] King R. J. (1975). Clinical relevance of steroid-receptor measurements in tumours.. Cancer Treat Rev.

[OCR_01034] Laing L., Smith M. G., Calman K. C., Smith D. C., Leake R. E. (1977). Nuclear oestrogen receptors and treatment of breast cancer.. Lancet.

[OCR_01052] McGuire W. L., Horwitz K. B., Pearson O. H., Segaloff A. (1977). Current status of estrogen and progesterone receptors in breast cancer.. Cancer.

[OCR_01058] Meyer J. S., Rao B. R., Stevens S. C., White W. L. (1977). Low incidence of estrogen receptor in breast carcinomas with rapid rates of cellular replication.. Cancer.

[OCR_01071] PEARSON O. H., RAY B. S. (1960). Hypophysectomy in the treatment of metastatic mammary cancer.. Am J Surg.

[OCR_01066] Panko W. B., MacLeod R. M. (1978). Uncharged nuclear receptors for estrogen in breast cancers.. Cancer Res.

[OCR_01076] Sheridan P. J., Buchanan J. M., Anselmo V. C., Martin P. M. (1979). Equilibrium: the intracellular distribution of steroid receptors.. Nature.

[OCR_01082] Stoll B. A. (1977). "False-positive" oestrogen-receptor assay in breast cancer.. Lancet.

[OCR_01085] Thorsen T. (1979). Occupied and unoccupied nuclear oestradiol receptor in human breast tumours: relation to oestradiol and progesterone cytosol receptors.. J Steroid Biochem.

[OCR_01091] Thorsen T., Stoa K. F. (1979). Nuclear uptake of oestradiol-17 beta in human mammary tumour tissue.. J Steroid Biochem.

[OCR_01096] Tisdale M. J. (1977). Inhibition of prostaglandin synthetase by anti-tumour agents.. Chem Biol Interact.

[OCR_01101] Traish A. M., Müller R. E., Wotiz H. H. (1979). Comparison of formation, activation, and nuclear translocation of receptor . estradiol (R . E2) complex at 0 degrees C and 37 degrees C in intact uterine cells.. J Biol Chem.

[OCR_01108] Williams D., Gorski J. (1971). A new assessment of subcellular distribution of bound estrogen in the uterus.. Biochem Biophys Res Commun.

